# Long-Term Adaption to High Osmotic Stress as a Tool for Improving Enological Characteristics in Industrial Wine Yeast

**DOI:** 10.3390/genes11050576

**Published:** 2020-05-20

**Authors:** Gabriela Betlej, Ewelina Bator, Bernadetta Oklejewicz, Leszek Potocki, Anna Górka, Magdalena Slowik-Borowiec, Wojciech Czarny, Wojciech Domka, Aleksandra Kwiatkowska

**Affiliations:** 1 Institute of Physical Culture Studies, College of Medical Sciences, University of Rzeszów, ul. Towarnickiego 3, 35-959 Rzeszów, Poland; gbetlej@ur.edu.pl (G.B.); ebator@ur.edu.pl (E.B.); wojciechczarny@wp.pl (W.C.); 2 Department of Biotechnology, College of Natural Sciences, University of Rzeszów, Pigonia 1, 35-310 Rzeszów, Poland; b.oklejewicz@gmail.com (B.O.); lpotok@o2.pl (L.P.); agorka@ur.edu.pl (A.G.); m.slowik_borowiec@interia.pl (M.S.-B.); 3 Medical College, University of Rzeszów, al. Rejtana 16c, 35-959 Rzeszów, Poland; w.domka@gazeta.pl

**Keywords:** industrial wine yeasts, high osmotic stress tolerance, long-term adaptation, adaptive laboratory evolution, resveratrol, resveratrol-enriched wine

## Abstract

Industrial wine yeasts owe their adaptability in constantly changing environments to a long evolutionary history that combines naturally occurring evolutionary events with human-enforced domestication. Among the many stressors associated with winemaking processes that have potentially detrimental impacts on yeast viability, growth, and fermentation performance are hyperosmolarity, high glucose concentrations at the beginning of fermentation, followed by the depletion of nutrients at the end of this process. Therefore, in this study, we subjected three widely used industrial wine yeasts to adaptive laboratory evolution under potassium chloride (KCl)-induced osmotic stress. At the end of the evolutionary experiment, we evaluated the tolerance to high osmotic stress of the evolved strains. All of the analyzed strains improved their fitness under high osmotic stress without worsening their economic characteristics, such as growth rate and viability. The evolved derivatives of two strains also gained the ability to accumulate glycogen, a readily mobilized storage form of glucose conferring enhanced viability and vitality of cells during prolonged nutrient deprivation. Moreover, laboratory-scale fermentation in grape juice showed that some of the KCl-evolved strains significantly enhanced glycerol synthesis and production of resveratrol-enriched wines, which in turn greatly improved the wine sensory profile. Altogether, these findings showed that long-term adaptations to osmotic stress can be an attractive approach to develop industrial yeasts.

## 1. Introduction

An evolutionary history of *Saccharomyces* wine yeasts includes spontaneous and naturally occurring events, such as heterozygosity, nucleotide and structural variations, introgressions, horizontal gene transfers, and hybridization, as well as human-enforced domestication effects [1,2,3,4]. Interestingly, this 7000-year-long history of tight interrelationships between yeast, humans, and human-related environments has resulted in the occurrence of genetic variants within some *Saccharomyces cerevisiae* lineages that are unique to several subpopulations (i.e., Japanese sake strains). The history of the domesticated *Saccharomyces* breeds is mainly linked to fermentation processes; hence, there is a history of the evolution of wine yeast toward coping strategies that address fermentation-related challenges [1]. This in turn has led to genetic divergence between wine and non-wine *Saccharomyces* strains [2].

Indeed, wine yeast cells are subjected to a variety of stresses during winemaking processes, such as high osmolarity, high sulfite dosages, nutrient depletion, acid stress, anaerobiosis, as well as a constantly increasing concentration of ethanol. The latter in turn exerts high chaotropic activity [5], reduces water activity [6] resulting in ethanol-induced water stress [7], and can negatively affect macromolecular systems [5]. However, as aforementioned, yeasts have been employed for wine production for thousands of years; therefore, they have evolved adaptive functions that enable them to fit in a fluctuating wine fermentation environment [1,2,3,8]. Such adaptations can be conferred by nucleotide changes, including base insertions, deletions, or substitutions, and/or by structural genome rearrangements on a large scale, such as chromosome duplications and translocations. These modifications can result in small-scale alterations in gene expression, protein structures, and protein interactions, or large-scale changes in genomic contexts or copy number variations [1,2,3,4].

Because of its extremely phenotypic plasticity and intra-specific variability, *S. cerevisiae* exhibits high competitive abilities, manifesting in its dominance within microbial communities in both spontaneous and inoculated ferments [1,8]. Collectively, these features resemble the characteristics and behavior of plant weeds [8]. The adaptability and competitive ability of *S. cerevisiae* underlie in turn an adaptive evolutionary-based approach, which is used by many researchers to develop industrial microorganisms with novel characteristics. In general, adaptive laboratory evolution (ALE) [9,10,11,12] is an approach based on the long-term adaptations of yeasts (and other organisms) to environmental and/or metabolic regimes. It is becoming an important alternative to strategies that utilize recombinant DNA technologies to develop economically important microorganisms [1,2,12]. Although genetic and/or genome engineering is nowadays more precise and has become a promising approach to develop wine yeast strains [1,2,12], consumers often do not accept the use of recombinant DNA technologies in food products [1,2,13,14].

The biotechnological usefulness of ALE strategy has been confirmed by several researchers to date, who employed the ALE approach to obtain thermotolerant [11], copper-tolerant [15], or saline-tolerant [16] yeast strains. In turn, the use of KCl-ALE (adaptive laboratory evolution under KCl-induced osmotic stress conditions) has been documented by Tilloy et al. [10], who successfully combined it with breeding approaches to remodel wine yeast strain metabolisms toward decreased ethanol production and enhanced glycerol biosynthesis [10]. The latter is thought to provide better sensorial of wine quality by improving its organoleptic properties, such as beverage body and fullness [17], as well as serves as a key osmoprotectant that preserves cells from the harmful effects of osmotic stress [18,19]. Besides, glycerol acts as a compatible solution that counteracts the negative effects of ethanol. The latter, which is concomitantly synthesized by *S. cerevisiae*, serves as an antimicrobial chaotrope that prevents the growth and metabolism of other microbial species [5].

Among other stress protectants that play a crucial role during winemaking processes is glycogen. It serves as the main carbon source and energy reserve as well as enhances cell viability under glucose deprivation conditions [8,20].

On the other hand, a high glucose concentration during fermentation affects the activity and stability of a wide range of yeast enzymes, for example, β-glucosidase [1,2,21]. The latter is an enzyme responsible for cleaving glycosylated precursors, i.e., piceid or polydatin, which are polyphenols found in grapes, into resveratrol [1,2,21]. Because of its antioxidant effects on the cardiovascular system [22], resveratrol has gained increasing public interest.

Both hyperosmolarity as well as a high glucose concentration or its deprivation are among the key parameters that are strictly linked to winemaking production, and they have a detrimental impact on yeast viability, growth, and fermentation performance [1,2,23]. Therefore, effective strategies that counteract the negative effects of specific enological conditions on yeast cells and/or improve the wine sensory profile are of high value for winemakers.

In this study, we subjected three industrial wine yeast strains to adaptive laboratory evolution under KCl-induced osmotic stress in order to develop wine yeast strains with increased fitness under high osmotic pressure as well as an increased ability for glycogen accumulation without the loss of their growth or fermentation abilities. Another aim of this study was to examine the levels of glycerol and resveratrol in wine products obtained with use of the KCl-evolved yeast strains.

## 2. Materials and Methods

### 2.1. Wine Yeast Strains, Growth Conditions, and Study Design

Three commercially available diploid wine yeast strains, previously described and characterized elsewhere [24,25] were used for this study. Briefly, strain 1 (trade name: Fermivin, species: *S. cerevisiae*) [24], strain 2 (trade name: Aromatic Wine Complex, species: *S. cerevisiae* var. *bayanus*) [24], and strain 3 (trade name: Complex IOC, species: *S. cerevisiae*) [25] were obtained from Biowin, Spirit ferm, and Mieszko, respectively

The ancestral yeast strains, each from one single colony, were cultured in liquid Yeast Extract Peptone Dextrose (YPD) medium (1% w/v Difco Yeast Extract, 2% w/v Difco Yeast Bacto-Peptone, 2% w/v dextrose) at 28°C. The cells were grown for 6 generations before being diluted (1:100) into fresh YPD medium. Yeast cells were cultured for 250 generations under standard conditions (control) or under increasing concentrations of KCl, which was added to the YPD medium. The KCl concentration began at 0.5 M for 50 generations, followed by 1 M for next 50 generations, and reached a final concentration of: a) 1.25 M, or b) 1.5 M KCl for the last 150 generations. The concentration of 1.5 M KCl was established as non-cytotoxic through previous research for the yeast strains investigated. 

Both ancestral strains and the corresponding YPD-evolved derivatives served as controls for the corresponding KCl-evolved strains. The YPD-evolved derivatives of the ancestral strains 1, 2, and 3 are denoted in this study as 1_YPD, 2_YPD, and 3_YPD, respectively. The 1.25 M KCl-evolved derivatives of strains 1, 2, and 3 are abbreviated here as: 1_KCl_a, 2_KCl_a, and 3_YPD_a, respectively. In turn, the 1.5 M KCl-evolved strains 1, 2, and 3 are denoted as: 1_KCl_b, 2_KCl_b, and 3_KCl_b, respectively.

At the end of the evolutionary experiment, samples of the ancestral and evolved (under standard conditions or in the presence of KCl) yeast strains were preserved as frozen (−80 °C) glycerol stocks. All analyses were performed with use of the yeast strains from glycerol stocks that were rescued in YPD agar plates (1% w/v Difco Yeast Extract, 2% w/v Difco Yeast Bacto-Peptone, 2% w/v dextrose, 2% agar) at 28 °C for 24 h and then grown under the same standard conditions (liquid YPD medium, at 28 °C) to enable the comparison and assessment of the features of the ancestral and evolved yeast cells.

### 2.2. Kinetics of Growth Assay

To determine growth kinetics, cells were washed, diluted (a) to a total volume of 150 μL with working solution of 5 × 106 cells/mL), and suspended in: a) YPD (1% w/v Difco Yeast Extract, 2% w/v Difco Yeast Bacto-Peptone, 2% w/v dextrose) medium (control), (b) YPD medium supplemented with 1.5 M KCl (osmotic stress), and (c) 30% YPGlu medium (1% w/v yeast extract, 2% w/v peptone, 30% w/v glucose) (high glucose stress). Then, they were cultured at 28 °C, and their growth was monitored turbidimetrically at 600 nm in a microplate reader every 1 h during a 16-h period. All experiments were performed in triplicate.

### 2.3. Cell Viability

Cell viability was evaluated using a LIVE/DEAD^®^ Yeast Viability Kit (Thermo Fisher Scientific, Warsaw, Poland) according to the manufacturer’s instructions. After staining with a mixture of FUN^®^ 1 and Calcofluor^®^ White M2R, cells were inspected under an Olympus BX61 fluorescence microscope equipped with a DP72 CCD camera and Olympus CellF software. For each analysis, 200 cells were used.

Cell viability was evaluated for both ancestral strains and their evolved derivatives in: (a) YPD medium (1% w/v Difco Yeast Extract, 2% w/v Difco Yeast Bacto-Peptone, 2% w/v dextrose), (b) YPD medium supplemented with 1.5 M KCl, and (c) 30% YPGlu medium (1% w/v yeast extract, 2% w/v peptone, 30% w/v glucose). Cell viability was assessed at the logarithmic phase of growth that was previously established for each growth condition. Cell viability was compared between the ancestral strains and their evolved derivatives separately for each growth condition variant.

### 2.4. Pulsed-Field Gel Electrophoresis (PFGE)

Preparation of agarose-embedded yeast DNA and PFGE separation of yeast DNA was performed according to Lewinska et al. [26].

### 2.5. Spot Test Assay

To evaluate the effect of KCl-induced osmotic stress on the growth phenotype of the analyzed strains, spot test assays were performed Briefly, serial dilutions of 1 × 10^6^, 1 × 10^5^, and 1 × 10^4^ cells mL-1 of a yeast exponential-phase culture (2 μL each) were spotted onto solid YPD medium (1% w/v Difco Yeast Extract, 2% w/v Difco Yeast Bacto-Peptone, 2% w/v dextrose), and, for stress tolerance analysis, onto solid YPD medium supplemented with 2 M KCl. Plates with yeast cells were incubated for 2 days at 28 °C, and then the growth of the evolved derivatives were compared to their corresponding ancestral strains. All experiments were performed in triplicate.

### 2.6. Iodine-Staining of Yeast Colonies

Yeast colonies were evaluated for glycogen storage as described elsewhere [27]. Briefly, both ancestral strains and the evolved derivatives were spotted onto: (a) Solid YPD medium (1% w/v Difco Yeast Extract, 2% w/v Difco Yeast Bacto-Peptone, 2% w/v dextrose, 2% agar), (b) YPD medium supplemented with 1.5 M KCl, and (c) 30% YPGlu medium (1% w/v yeast extract, 2% w/v peptone, 30% w/v glucose). Plates with yeast cells were incubated for 48, 72, and 96 h at 28 °C, and then stained for 1 min with iodine (0.2% I2 in 0.4% KI). All experiments were performed in triplicate.

### 2.7. Laboratory-Scale Fermentations and Measurements of Glucose, Fructose, Glycerol, and Resveratrol Concentrations

Laboratory-scale fermentation in grape juice was performed as previously described [28] with small modifications. Briefly, yeasts from a single colony were inoculated into 5 mL of YPD medium (1% w/v Difco Yeast Extract, 2% w/v Difco Yeast Bacto-Peptone, 2% w/v dextrose) and grown for 16 h at 28 °C. For the fermentation experiment, the 2.5 × 106 yeast cells mL-1 of the yeast strain pre-cultures were inoculated into flasks containing 100 mL of sterile commercially available grape juice (Rabenhorst, Germany) containig 18% sugars (10.2 g/L glucose and 7.7 g/L fructose), or 30% sugars (15.82 g/L glucose; 13.55 g/L fructose). Fermentation was conducted at 25 °C with shaking (100 rpm.) for 21 days.

The concentrations of glucose, fructose, glycerol, and resveratrol in the media were determined by HPLC at the end of the fermentation process. All experiments were performed in triplicate. Data are presented as the means ± SD. Statistical significance was assessed by one-way ANOVA and with the Tukey’s multiple comparison test. Significance was accepted at *p* < 0.05.

### 2.8. HPLC

All experiments were performed on an HPLC Ultimate 3000 system (Dionex, Germering, Germany) consisting of a charged aerosol detector ultra RS (ESA, Chelmsford, MA, USA). Data processing was carried out with Chromeleon 6.8 software (Dionex). Chromatographic analysis was carried out at 35 °C. Samples were separated on a Shodex Ashipack NH2P-50 column packed with 5-μm shell particles (250 mm × 4.6 mm) and acetonitrile–water (75/25% v/v) mobile phase at a flow rate of 1.50 mL/min. The nitrogen (99.99%) gas flow rate was regulated automatically at 35 psi and monitored by the CAD device. The total time of analysis amounted to 20 min. HPLC-grade acetonitrile was obtained from J.T.Baker (Malinckrodt Baker B.V., Holland). The linearity of the detector response was determined by the square correlation coefficients of the calibration curves generated by injections of standard solutions. The range of linearity was established by injecting six different concentrations obtained by the dilution of a standard mixture of sugars and resveratrol in ultrapure water and methanol, respectively. Linearity was studied between 0.02 and 20 g/L and showed coefficients of determination (R2) ≥ 0.99 for all substances. The limit of quantification (LOQ) and limit of detection (LOD) for test substances were 0.02 and 0.01 g/L, respectively. The wine samples were prepared for analysis by centrifugation (10,000 rpm, time 10 min) and filtered through 0.45-um filter couplers PTFE (Millipore, Poland).

### 2.9. Semi-Quantitative PCR

The total RNA from the cells was isolated using a Universal RNA Purification Kit (EURx, Poland), treated with TURBO™ DNAse (Thermo Fisher Scientific, Warsaw, Poland), and reverse transcribed with the use of an iScript™ Select cDNA Synthesis Kit (Bio-Rad, Warsaw, Poland) according to the manufacturer’s instructions. Semi-quantitative PCR were performed using OptiTaq PCR Master Mix (EURx, Poland) according to the manufacturer’s protocol with *S. cerevisieae* gene-specific primers: HOG1L: 5’-TTGACATCTCAGCCAGTTGC-3’ and HOG1P: 5’-TTTCCAAGGGTCTTGTTTGC-3’ for *Hog1*, GPD1L: 5’-TTTTGCCCCGTATCTGTAGC-3’ and GPD1P: 5’-CTCGCCTCTGAAATCCTTTG-3’ for *Gpd1*, GPD2L: 5’-TTTCCCAGAATCCAAAGTCG-3’ and GPD2P: 5’-CGGATTGACCGTTAAGCAAT-3’ for *Gpd2*, GPP1L: 5’-GCCAAGAAATGGTTCGACAT-3’ and GPP1P: 5’-CAACGATTTTACAGCCAGCA-3’ for *Gpp1*, GPP2L: 5’-GGTGCAGTTAAGCTGTGCAA-3’ and GPP2P: 5’-CCATTCCTGCCCTTCAGATA-3’ for *Gpp2*, as well as ActL: 5’-GCCTTCTACGTTTCCATCCA-3’ and ActP: 5’-GGCCAAATCGATTCTCAAAA-3’ for the actin gene. The PCR conditions were as follows: Denaturation at 95 °C for 5 min; 27–30 cycles of 95 °C for 30 s; 58 °C for 30 s for Hog1, Gpp1, and actin; 61 °C for 30 s for Gpp2 and Gpd2; or 62 °C for 30 s for Gpd1, followed by 72 °C for 30 s. The optimal amount of cDNA and the optimal number of cycles were determined for each primer pair to ensure the linearity of the PCR reaction. The expression of actin was used as a control. All experiments were performed in triplicate.

## 3. Results and Discussion

### 3.1. Wine Yeast Strain Screening and Characterization

Previously, we screened and characterized 17 industrial wine yeast strains for their response to generational and ethanol-mediated changes [24,25], and we chose three of them for this study. These three strains were chosen because they were: (a) Widely used in winemaking, (b) well-characterized at a molecular level, as well as (c) being the most prone to generational changes [24,25]. Array-CGH profiles of strains 1 and 2 revealed that the most significant diversity in the gene copy number was observed within sub-telomeric regions in almost all chromosomes investigated; there was also a generation-mediated gain of genes responsible for telomere maintenance (*YRF1* genes) and copper and cadmium detoxification (*CUP1* genes) [24]. Such copy number variations of *CUP1* are domestication-related traits in wine yeasts [29,30]. Similarly, plasticity of the telomeric regions, reflected by several chromosomal translocations, is often observed in industrial yeast strains [1,2,3,4,31].

Analysis of the strain 3 genome using next-generation sequencing revealed that this strain showed 47,575 high-quality variants, including single nucleotide variants and indels, compared to the reference S288C genome. Almost 60% of the affected genes were primarily involved in metabolic process, in particular protein metabolism and DNA/RNA metabolic processes [17]. In addition, GSH analysis showed generational and ethanol-mediated changes in strain 3, which were manifested by an overrepresentation of genes belonging to positive transcription regulators, when compared to the corresponding ancestral strain [25].

In this study, we subjected the abovementioned strains to KCl-induced osmotic stress for 250 generations. Although KCl is not present in grape must, it serves as an osmotic stressor that is widely used as a model for studies of osmotic stress in fungi [10,19,32,33].

**Figure 1:**
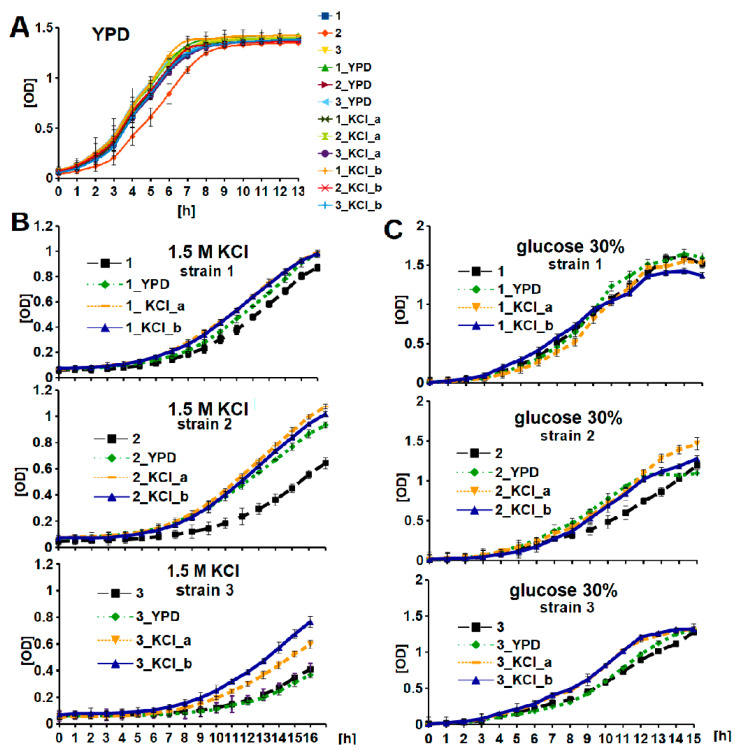
Generational and osmotic stress-induced changes in the growth rate of strains 1, 2, and 3. Ancestral and evolved yeast strains were grown in (**A**) YPD medium under standard conditions; (**B**) in the presence of 1.5 M KCl; or (**C**) high glucose concentration. Data are presented as the means ± SD; n = 3. 1, 2, and 3–ancestral strains 1, 2, and 3, respectively; 1_YPD, 2_YPD, 3_YPD–YPD-evolved derivatives of strains 1, 2, and 3 (control), respectively; 1_KCl_a, 2_KCl_a, and 3_KCl_a–1.25 M KCl-evolved derivatives of strains 1, 2, and 3, respectively; 1_KCl_b, 2_KCl_b, and 3_KCl_b–1.5 M KCl-evolved derivatives of strains 1, 2, and 3, respectively.

Because it is widely accepted that high osmotic pressure may affect the growth and viability of the yeast cells as well as the genome stability [20,24,25], these three characteristics were determined. Although, there were no changes in growth when yeast strains were grown in standard YPD medium, strain-dependent alterations in growth were observed under stress conditions. Accelerations of growth were observed for three evolved derivatives of strain 2 (including the control 2_YPD strain) and for two KCl-evolved derivatives of strain 3 in the presence of both 1.5 M KCl and a high glucose concentration. Such an effect was not observed in the case of strain 1 (Figure 1).

Changes in the growth rate did not result from cell death as cell viability was not compromised (data not presented).

Because genetic stability is one of the desirable characteristics of wine yeasts [34], we compared the chromosome patterns between evolved and the corresponding ancestral strains. Pulsed-field gel electrophoresis (PFGE) analysis revealed that adaptive evolution under both control and KCl-induced osmotic stress did not affect the karyotypes of the analyzed strains (data not presented).

Amongst other desirable features of wine yeast strains are high tolerance to osmotic stress as well as enhanced vitality. The latter term refers to the metabolic activity, fitness, or vigor of a starter culture and can be indirectly evaluated by measuring, i.e., storage molecules, including glycogen [34]. Therefore, both the tolerance to high osmotic pressure and the ability of the evolved yeast strains to accumulate glycogen were assessed.

### 3.2. KCl-ALE-Based Improvement of Wine Yeasts’ Tolerance to Osmotic Stress and Glycogen Accumulation

**Figure 2:**
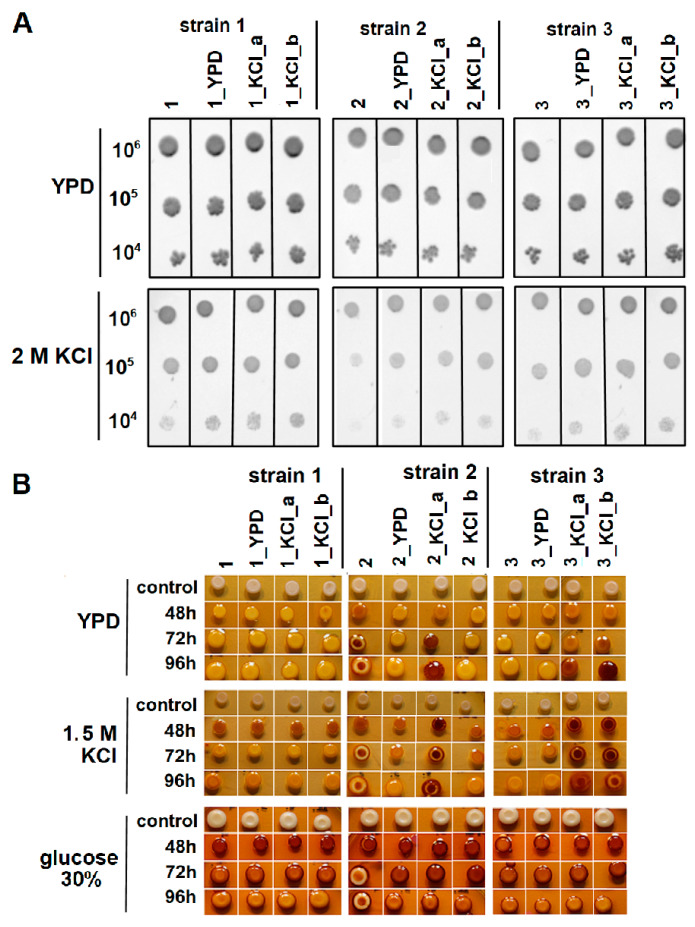
(**A**) Analysis of the effect of KCl-induced osmotic stress on the growth phenotype of the analyzed strains by spot test assays. Yeast cells were grown on solid YPD medium (control) or on YPD medium supplemented with 2 M KCl (osmotic stress conditions). Representative photographs are shown. (**B**) Iodine-staining of glycogen stores (indicated by reddish-brown coloration) of yeast colonies. Ancestral and evolved derivatives of strains 1, 2, and 3 were grown for 48, 72, and 96 h in standard solid YPD medium, YPD medium supplemented with 1.5 M KCl, or YPGlu containing 30% glucose. The staining reactions of the colonies were recorded for 1 min after adding the iodine solution. Control-yeast colonies not treated with iodine solution. 1, 2, and 3–ancestral strains 1, 2, and 3, respectively; 1_YPD, 2_YPD, 3_YPD – YPD-evolved derivatives of strains 1, 2, and 3 (control), respectively; 1_KCl_a, 2_KCl_a, and 3_KCl_a–1.25 M KCl-evolved derivatives of strains 1, 2, and 3, respectively; 1_KCl_b, 2_KCl_b, and 3_KCl_b–1.5 M KCl-evolved derivatives of strains 1, 2, and 3, respectively.

After the end of the ALE experiment, we assessed an effect of 2M KCl-induced osmotic stress on the growth phenotype of the analyzed strains by using a spot test assay, which is a standard method to evaluate the growth phenotype [35,36,37]. We showed that KCl-evolved derivatives of strains 2 and 3 (2_KCl_a, 2_KCl_b as well as 3_KCl_a, and 3_KCl_b, respectively) significantly increased their fitness to high osmotic pressure. Interestingly, this effect was also generational dependent because enhanced tolerance to high osmotic stress was also observed for two derivatives of strains 2 and 3 that were cultured for 250 generations under standard conditions (2_YPD and 3_YPD, respectively) (Figure 2A). In contrast to strains 2 and 3, evolved derivatives of strain 1 showed only slight increases in tolerance to high osmotic stress, when compared to the corresponding ancestral strain (Figure 2A)

Similarly, adaptive evolution under osmotic stress conditions had an impact on strains 2 and 3 with respect to the ability for glycogen accumulation. This effect was observed for 2_KCl_a, 3_KCl_a, and 3_KCl_b strains throughout the 96 h of growth under control conditions and in the presence of 1.5 M KCl as was indicated by the reddish-brown coloration of yeast colonies after iodine staining (Figure 2B). Although the impact of glycogen on the internal osmotic pressure of the cell is low, this macromolecule plays a crucial role in cells’ survival during prolonged nutrient deprivation. Therefore, yeast cells with an increased ability for glycogen accumulation show enhanced fitness and vitality [20,38,39]. Indeed, cells of *Sacchaormyces* accumulate storage carbohydrates very efficiently, as glycogen along with trehalose can reach 25% dry weight [8]. From the winemaking point of view, yeast cells overproducing glycogen are desirable, because they display improved performance upon inoculation into grape juice [32] and are more tolerant to different enological-related stresses. For example, it has been shown that yeast strains overexpressing *GSY2*, encoding glycogen synthase, exhibited enhanced viability under both laboratory growth and fermentation conditions in synthetic and natural musts. In particular, they showed increased resistance to glucose deprivation conditions [20]. It should be mentioned that we observed enhanced glycogen accumulation in all evolved derivatives of strain 2 under high glucose concentration conditions, when compared to its corresponding ancestral strain. Although there were no differences in glycogen accumulation over 48 h, significant increases in its content were observed at 72 and 96 h of growth (Figure 2B). Perhaps, changes in glycogen accumulation, mediated by adaptive evolution, might be explained by the fact that glycogen metabolism is strongly affected by a wide variety of cellular processes, including osmotic stress [39].

### 3.3. Adaptive Evolution-Mediated Changes in Glycerol Concentrations

Because Tilloy et al. [10] used the ALE-KCl strategy to remodel wine yeast strain metabolisms toward decreased ethanol production and enhanced glycerol biosynthesis [10], the next question we asked was whether KCl-evolved strains redirected their carbon flux towards glycerol overproduction.

**Figure 3:**
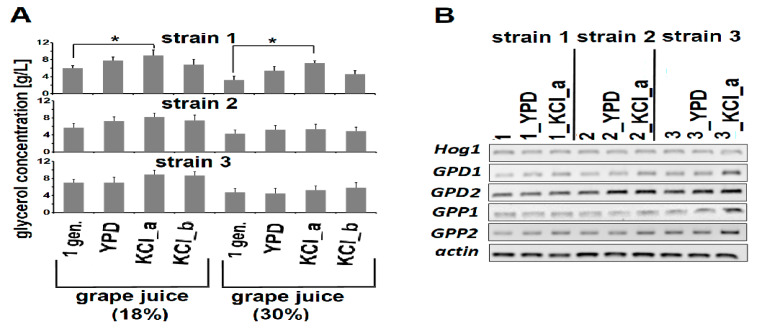
(**A**) Concentrations of glycerol [g/L] after the end of fermentation. Grape juice containing 18% sugars (10.2 g/L glucose and 7.7 g/L fructose) or 30% sugars (15.82 g/L glucose; 13.55 g/L fructose) were used as fermentation media. Data are presented as the means ± SD; *n* = 3. Significant differences are marked by * (at *p* < 0.05). 1 gen. –1st generation (ancestral strains); YPD–YPD-evolved derivatives (control); KCl_a–1.25 M KCl-evolved derivatives; KCl_b–1.5 M KCl-evolved derivatives. (**B**) Semi-quantitative PCR analysis of *Hog1*, *GPD1*, *GPD2*, *GPP1*, and *GPP2* expression levels determined for ancestral and evolved yeast strains 1, 2, and 3. Actin was used as an internal control. Representative gels are shown. **1**, **2**, and **3**–ancestral strains 1, 2, and 3, respectively; 1_YPD, 2_YPD, 3_YPD–YPD-evolved derivatives of strains 1, 2, and 3 (control), respectively; 1_KCl_a, 2_KCl_a, and 3_KCl_a–1.25 M KCl-evolved derivatives of strains 1, 2, and 3, respectively; 1_KCl_b, 2_KCl_b, and 3_KCl_b–1.5 M KCl-evolved derivatives of strains 1, 2, and 3, respectively.

Therefore, we performed a laboratory-scale fermentation in grape juice containing 18% or 30% sugars. At 21 days of fermentation, when sugars (glucose and fructose) in the media were exhausted, concentrations of glycerol in the media were determined. The highest increase in glycerol production was exhibited by one of the KCl-evolved derivatives of strain 1 (1_KCl_a strain), while KCl-evolved derivatives of strain 3 (3_KCl_a and 2_KCl_b) showed the lowest rises in glycerol synthesis, in comparison to the corresponding ancestral strains (Figure 3A).

When 18% grape juice was used as fermentation media, concentrations of glycerol were 5.97 (± 0.62), 7.8 (± 0.82), 8.96 (± 1.32), and 6.86 (± 1.21) g/L for strains 1, 1_YPD, 1_KCl_a, and 1_KCl_b, respectively. In turn, for strains 3, 3_YPD, 3_KCl_a, and 3_KCl_b, concentrations of glycerol were 7.02 (± 0.78), 7.04(± 1.25), 8.89 (± 1.03), and 8.65 (± 0.93) g/L, respectively. In the case of strain 2, glycerol concentrations reached 5.68 (± 1.02), 7.24 (± 0.98), 8.24 (± 0.87), and 7.42 (± 1.24) g/L for strains 2, 2_YPD, 2_KCl_a, and 2_KCl_b, respectively (Figure 3A).

Similarly, when fermentation was conducted in grape juice containing 30% sugars, the highest increase in glycerol production was observed for the strain 1_KCl_a, a derivative of strain 1, when compared to its ancestral strain. The lowest rises in glycerol content were observed when 30% grape juice was inoculated with KCl-evolved derivatives of strain 3. At the end of fermentation, the glycerol concentration reached 3.2 (±0.92), 5.43 (±0.99), 7.2 (±0.52), and 4.6 (±0.82) g/L for strains 1, 1_YPD, 1_KCl_a, and 1_KCl_b, respectively; 4.31 (±0.86), 5.2 (±0.99), 5.32 (±1.2), and 4.9 (±0.91) g/L for strains 2, 2_YPD, 2_KCl_a, and 2_KCl_b, respectively; and 4.76 (±0.89), 4.46 (±1.2), 5.26 (±0.99), and 5.81 (±1.2) g/L, respectively (Figure 3A).

Our results showing adaptive evolution-mediated increases in glycerol production are consistent with data obtained by Tilloy et al. [10], who confirmed that the KCl-ALE approach is an effective strategy to remodel yeast metabolism towards enhanced glycerol production and decreased ethanol biosynthesis [10]. However, it should be mentioned that the authors [10] evolved their yeast strain for 450 generations (in contrast to 250 generations in our study), and they implemented a breeding approach in addition to KCl-ALE [10].

It is widely known that intracellular glycerol is a key cellular osmoprotectant [40]. However, it is also accepted that the glycerol content is regulated by several mechanisms, such as metabolic reprogramming of carbon fluxes, control of glycerol export and import, as well as an adjustment of glycerol biosynthesis-related gene expression [17]. In general, the intracellular concentration of glycerol increases in response to high osmotic stress via the high osmolarity glycerol (HOG) pathway. Its central kinase, Hog1p, is activated by phosphorylation and activates, i.e., the expression of genes responsible for glycerol uptake and synthesis [17,41]. Gene expression can be adapted evolutionarily to stress, e.g., salt stress. Dhar et al. [16] showed that yeast cells subjected to NaCl treatment for 300 generations achieved evolutionary adaptation to saline, which, in turn, was associated with both a genome size increase and modest expression changes in several genes, including stress-responsive ones, for example, *Msn4* (multicopy suppressor of SNF1 mutation), encoding a stress-inducible transcriptional activator [16].

Therefore, the next question that we asked was whether an enhanced tolerance of high osmotic stress as well as the increased levels of glycerol synthesis exhibited by the evolved strain might be linked to any changes in osmoresponsive gene expression. To answer this question, all investigated samples were collected after the end of the evolutionary experiment and were analyzed for their glycerol biosynthesis-related gene expressions.

Among the osmoresponsive genes that are responsible for glycerol production and whose expressions are partially Hog1 dependent are two paralogs of NAD+-dependent glycerol-3-phosphate dehydrogenases (GPDs), that is *GPD1* and *GPD2* [40,41,42], as well as two paralogs of specific phosphatases (GPPs), that is *GPP1* and *GPP2* [41]. The action of GPDs leads to the reduction of the glycolytic intermediate di-hydroxyl-acetone phosphate (DHAP) to glycerol-3-phosphate (G3P) [42,43,44], which in turn is dephosphorylated by GPPs [41]. However, semi-quantitative RT-PCR analysis showed only slight increases in *GPD1*, *GPP*1, and *GPP2* expression levels in the case of the KCl-evolved strain 3 (3_KCl_a strain), when compared to its ancestral strain (Figure 3B).

Nevertheless, it has been discussed whether all four genes played equally important roles in osmoadaptation. For example, Babazadeh et al. [41], who revised data concerning the osmoadaptation mechanisms of yeast cells, reported that such a successful adaptation is conferred by the upregulation of only *GPD1* and *GPP2* [41]. In support of this, there are data showing that expression of their paralogs, *GPD2* [45] and *GPP1* [43], respectively, are also influenced by other stresses, such as hypoxia [43,45]. In addition, Remize et al. [46] showed that a major role in glycerol production is played by GPD1, which is only partially controlled by the HOG1 pathway, while the role of *GPD2* during anaerobic fermentation was found to be of little significance [46].

Therefore, the enhanced levels of *GPD1* and *GPP2* as well as *GPP1* transcripts noticed in the 3_YPD_KCl_a (Figure 3B) might result not only from hyperosmolarity but also from hypoxic stress, which is associated with the fermentation process. In addition, such an observation might confirm the data obtained by Dhar et al. [47], who showed that yeast coped with stressful changing environments by cross-protection, which is a situation when one environmental stressor protects cells against a second stressor [48]. Indeed, recent studies on *S. cerevisiae* adaptations have shown enhanced stress tolerance after long-term exposure to glycerol [49] even though glycerol does not cause osmotic stress [19]. One of these mechanisms that has been recognized as a physiological adaptation by yeast cells to cope with changing environments is an increase in genome-scale gene expression for short durations [47], which can also be mediated by duplicated genes [49]. Interestingly, changes in gene expression may be strictly limited to genes engaged in response to a specific stressor or responsible for coping with an anticipated environmental agent linked to the present stimulus [47].

However, the molecular basis for the enhanced mRNA levels observed in this study remains to be elucidated, since they could have resulted from increased transcription rates, transcript stabilizations, or both. In fact, there are several reports showing that the mRNAs of genes engaged in the stress response are regulated differently in comparison to other [50,51]. Examples include the upregulation of synthesis, the extension of the transcript half-lives [36], and finally selective mRNAs stabilization (or degradation) that, in turn, are dependent on the stress response phase [51].

### 3.4. Adaptive Evolution-Mediated Changes in Resveratrol Concentrations

Besides high tolerance to osmotic stress, enhanced levels of intracellular glycogen, or increased production of glycerol, another desirable feature of wine yeast strains is the ability to liberate glycosylated flavor precursors [2]. The latter greatly improves the sensory profile of wine and, in addition, some of them may be beneficial for health. For example, resveratrol (3,5,4′-trihydroxystilbene or 3,5,4′-stilbenetriol), which belongs to plant stilbenes and is synthesized in the skin cells of grape berries, is a well-known antioxidant reducing the risk of coronary heart disease [22,32]. Resveratrol is also found in red wine because of skin contact during the first phase of fermentation [32]. Nevertheless, resveratrol in grape berries occurs mainly in two glycosylated forms, known as piceid or polydatin, which, however, are more hydrophilic and less bioavailable to humans than resveratrol [21]. Glycosylated forms of resveratrol can be hydrolyzed due to the activity of β-glucosidase, which in turn is found in some wine-related yeast strains [52,53].

Therefore, at the end of fermentation, we determined the resveratrol concentrations in fermentation media. The next question we asked was whether adaptive evolution-mediated changes might affect yeast strains towards the production of resveratrol-enriched wines.

**Figure 4:**
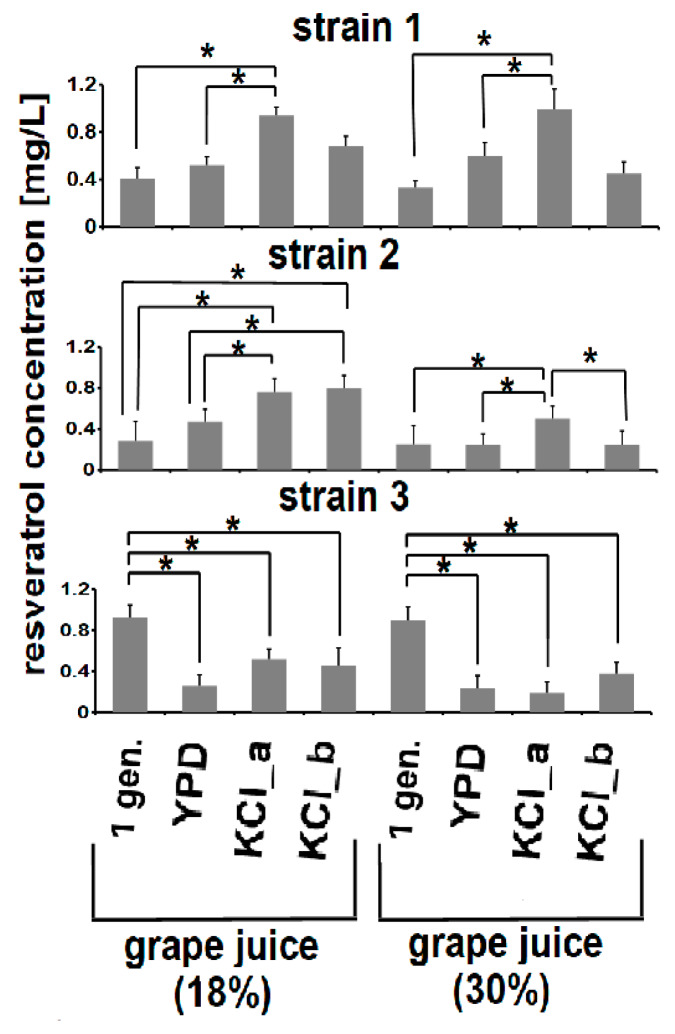
Concentrations of resveratrol [mg/L] after the end of fermentation. Grape juice containing 18% sugars (10.2 g/L glucose and 7.7 g/L fructose) or 30% sugars (15.82 g/L glucose; 13.55 g/L fructose) were used as fermentation media. Data are presented as the means ± SD; *n* = 3. Significant differences are marked by * (at *p* < 0.05). 1 gen—1st generation (ancestral strains); YPD–YPD-evolved derivatives (control); KCl_a–1.25 M KCl-evolved derivatives; KCl_b–1.5 M KCl-evolved derivatives.

First, we found that changes in resveratrol concentrations in the fermentation media were strain dependent. Second, adaptive evolution under KCl-induced osmotic stress had an impact on yeast strains with regard to changes in the resveratrol content in the fermented grape juice. Significant increases in resveratrol concentrations were observed, when grape juice was inoculated with the evolved derivatives of strains 1 and 2, in comparison to the corresponding ancestral strains (Figure 4). At the end of the 18% grape juice fermentation, the resveratrol concentrations were 0.41 (±0.09), 0.52 (± 0.07), 0.94 (± 0.07), and 0.68 (± 0.09) mg/L for strains 1, 1_YPD, 1_KCl_a, and 1_KCl-b, respectively; and 0.28 (± 0.01), 0.47 (± 0.02), 0.76 (± 0.01), and 0.8 (± 0.03), for strains 2, 2_YPD, 2_KCl_a, and 2_KCl_b, respectively (Figure 4).

Concentrations of resveratrol in 30% grape juice inoculated with strains 1, 1_YPD, 1_KCl_a, and 1_KCl-b were 0.33 (± 0.06), 0.60 (± 0.02), 0.99 (± 0.01), and 0.45 (± 0.02), respectively; and for strains 2_YPD, 2_KCl_a, and 2_KCl_b were 0.25 (± 0.02), 0.24 (± 0,01), 0.5 (± 0.01), and 0.24 (± 0.01) mg/L, respectively (Figure 4).

In contrast, resveratrol concentrations were significantly decreased in the case of inoculations of grape juice with evolved derivatives of strain 3, when compared to its ancestral strain. The resveratrol concentration reached 0.93 (± 0.01) and 0.9 (± 0.01) mg/L for strain 3 growing in 18% and 30% grape juice, respectively. Decreases in its content were observed for strains 3_YPD (0.26 ± 0.01 mg/L), 3_KCl_a (0.52 ± 0.02 mg/L), and 3_KCl-b (0.46 ± 0.02) growing in 18% grape juice as well as for 3_YPD (0.24 ± 0.01 mg/L), 3_KCl_a (0.19 ± 0.02 mg/L), and 3_KCl-b (0.38 ± 0.02) growing in 30% grape juice (Figure 4).

In general, many desirable enzymes, including β-glucosidases, which are responsible for modulating compounds of the grape must (by producing or releasing the preferred compounds from their precursors) and thereby affecting composition of wines, are found mainly in non-*Saccharomyces* yeasts [1,2]. Therefore, non-*Sacchorymyces* yeasts are often incorporated into mixed-culture fermentations; non-*Sacchorymyces* yeasts, in cooperation with *Saccharomyces* spp., greatly improve the chemical composition and sensory profile of wines [1,2].

To date, several strategies have been developed to obtain resveratrol-enriched wines. Some of these approaches focus on developing genetically modified wine yeasts that will be able to produce resveratrol during fermentation. For example, Becker et al. [54] made an attempt to reconstruct a biochemical pathway in a heterologous host to produce resveratrol [54]. Other strategies are aimed at selecting the best β-glucosidase-producer yeasts that are able to hydrolyze glycosylated forms of resveratrol under enological conditions [52,53]. For example, in one study, an extracellular glucan β-glusosidase precursor was identified and isolated from *Dekkera bruxellensis* wine yeast [55]. This enzyme consists of two subunits, SCW4p and glucan β-glucosidase precursor, although only the latter subunit has been shown to possess extracellular β-glucosidase activity that was responsible for the conversion of piceid from *Polygonum cuspidatum* into resveratrol [55]. Nevertheless, *D. bruxellensis* is generally considered the main spoilage yeast during winemaking due to the production of specific compounds like various pungent volatile phenols, associated with unpleasant aromas, which are collectively known as the phenolic taint or “brett” flavor [1,2]. Therefore, in another study, 308 autochthonous yeast strains isolated from *Vitis labrusca* Bordô grapes were screened for β-glucosidase production; 33 out of the 308 analyzed strains were reported to be able to hydrolyze piceid into free resveratrol in synthetic media (along with the ability to hydrolyze other substrates, such as cellobiose and arbutin and/or esculin) [52]. Among them were strains from the following yeast species: *Hanseniaspora uvarum* (*n* = 23), *Hanseniaspora opuntiae* (*n* = 4), *Candida zemplinina* (*n* = 3), *S. cerevisiae* (*n* = 1), *Zygoascus meyerae* (*n* = 1), and *Zygosaccharomyces bailii* (*n* = 1). With regard to *S. cerevisiae*, it also should be mentioned that only one strain out of 11 was able to hydrolyze piceid into resveratrol due to β-glucosidase activity [52].

Here, we showed that adaptive evolution under osmotic stress can be a useful approach to develop yeast strains towards the production of wines with an increased content of resveratrol. Of course, there are some strain-dependent limitations. For example, the evolved derivatives of strain 3 failed to produce resveratrol-enriched wines. In fact, β-glucosidase activity, which is responsible for cleaving the glycosylated precursors of resveratrol, can be significantly decreased and/or inhibited under winemaking conditions, such as, i.e., low pH value, high initial glucose concentration, and high ethanol concentration [53]. On the other hand, there are yeasts that produce β-glucosidases whose enzymatic activity and stability are not affected by enological conditions. These are not repressed by a high concentration of glucose and are resistant to ethanol and a low pH [21]. Perhaps, strains 1 and 2 are producers of such β-glucosidases. The findings showing that KCl-evolved strains 1 and 2 are more efficient in producing resveratrol-enriched wines in comparison to the corresponding ancestral strains, which might result from adaptive evolution-mediated changes in protein structures [3,4], including β-glucosidases, that affect their activity and/or stability under enological conditions. As subtle changes in protein structures might result from adaptive evolution-driven mutations, one can assume that our suggestions are in line with the results obtained by Kuo et al. [55]. Kuo et al. [55] showed that 12 out of 226 isolates of *D. bruxellensis* wine yeast, which previously had been subjected to the *N*-methyl-N0-nitro-N-nitrosoguanidine (NTG) mutagenesis, exhibited enhanced β-glucosidase activity towards piceid conversion into resveratrol [55].

Nevertheless, it also should be taken into consideration that many industrial relevant traits and performance parameters are polygenic and are not always recognized as being related to the particular trait. Besides, each biological system is the whole, which is greater than a sum of its parts. Due to these emergent properties, which result from numerous interaction effects between different organism elements, the final output of, i.e., metabolic engineering/reprogramming can be difficult or even impossible to predict [12]. This is one of the most important challenges that many approaches, including synthetic biology, have to face [1,12].

## 4. Conclusions

The main aim of this study was to develop wine yeast strains with increased tolerance to high osmotic stress, enhanced ability to accumulate glycogen, as well as augmented capability to produce glycerol and resveratrol. All of these characteristics are desirable wine yeast properties that can greatly improve both the fermentation process as well as the sensory profile of wine. To achieve these goals, we employed adaptive evolution under osmotic stress and showed that (i) strains 2 and 3 greatly enhanced their tolerance to high osmotic stress, (ii) the evolved derivatives of strains 2 and 3 gained the ability to accumulate glycogen, (iii) strain 1 enhanced the synthesis of glycerol, while (iv) the evolved derivatives of strains 1 and 2 were able to produce resveratrol-enriched wines. Of course, it cannot be excluded that some of these adaptive-mediated changes might be stochastic and/or strongly depend on the genetic background of the individual strains. However, such an approach might be an attractive strategy to develop industrial wine yeast strains with desirable characteristics.
